# Characteristic Evaluation of an Intensifier Detector for SMILE UVI

**DOI:** 10.3390/s26020483

**Published:** 2026-01-11

**Authors:** Yongmei Wang, Xiaohong Liu, Pengda Li, Jinghua Mao, Weipeng Huang, Guojun Du, Ziyue Wang, Zhuo Zhang, Sylvain VEY, Rene Berlich, Fei He

**Affiliations:** 1 National Space Science Center, Chinese Academy of Sciences, Beijing 100190, China; liuxiaohong@nssc.ac.cn (X.L.); lipengda@nssc.ac.cn (P.L.); maojinghua@nssc.ac.cn (J.M.); huangweipeng@nssc.ac.cn (W.H.); wangziyue@nssc.ac.cn (Z.W.); zhangzhuo@nssc.ac.cn (Z.Z.); 2 School of Astronomy and Space Science, University of Chinese Academy of Sciences, Beijing 100049, China; 3 University of Chinese Academy of Sciences, Beijing 100049, China; 4 Beijing Institute of Space Mechanics & Electricity, Beijing 100194, China; jun432432@sina.com; 5 European Space Research and Technology Centre, European Space Agency, 2200 AG Noordwijk, The Netherlands; sylvain.vey@esa.int (S.V.); rene.berlich@esa.int (R.B.); 6 Key Laboratory of Earth and Planetary Physics, Institute of Geology and Geophysics, Chinese Academy of Sciences, Beijing 100029, China; hefei@mail.iggcas.ac.cn; 7 College of Earth and Planetary Sciences, University of Chinese Academy of Sciences, Beijing 101408, China

**Keywords:** image intensifier, ICCD, relay lens system, UVI, FUV

## Abstract

As one of the payloads on board the Solar wind Magnetosphere Ionosphere Link Explorer (SMILE) spacecraft, the ultraviolet imager (UVI) aims to capture N2 Lyman–Birge–Hopfield (LBH) aurora continuously on a high-eccentricity orbit. The UVI instrument includes an intensified charge-coupled device (ICCD) for far ultraviolet (FUV) wavelength. It comprises a sealed image intensifier, a relay lens system, a CCD, and a mechanical housing. ICCD’s performance characteristics are evaluated before integrating with the optical system of the UVI, including the quantum efficiency, radiant gain, background characteristics, excess noise factor, image quality, and signal-to-noise ratio (SNR). The testing procedure and results are presented and discussed. The results demonstrate that the comprehensive performance of the detector is good, and provide critical technical support for quantitative applications.

## 1. Introduction

SMILE is a new project that employs advanced identification methods to simultaneously acquire images and movies of the magnetopause, cusps, and auroral oval, thereby revealing the physics of the magnetosphere [1]. SMILE is a common space science mission between the European Space Agency (ESA) and the Chinese Academy of Sciences (CAS). As one of the four payloads aboard the SMILE mission, the ultraviolet imager (UVI) aims to capture the size of the polar cap, the auroral oval’s position and width, and the transient and localized brightening occurring within and on the auroral oval edges. It provides an unprecedented opportunity for continuous and real-time monitoring of the Northern Aurora on the SMILE mission.

FUV imaging is the primary method for obtaining global, highly sensitive observations of auroras in space. To overcome the limitations of visible-light observations caused by solar illumination pollution, it uses auroral radiation in the FUV band (main characteristic spectral lines such as OI 135.6 nm, 130.4 nm, and the N_2_ LBH band), thereby achieving all-day, global, and highly sensitive aurora monitoring. Using high-efficiency wavelength-selecting technology, and combining the large-area array two-dimensional imaging detector with an image intensifier directly led to a leap in imaging performance, enabling two-dimensional, global, and instantaneous morphology imaging of weak FUV signals. The Spectrographic Imager (SI) and the Far Ultraviolet Wideband Imaging Camera (WIC) instruments on board the IMAGE and POLAR/UVI demonstrated good aurora-imaging capabilities in the day side, acquiring continuous aurora observation for less than 10 h [2,3].

The SMILE mission operates in a highly eccentric orbit with an orbital period of approximately 50 h. It helps UVI to produce an uninterrupted image of the auroral oval with a 1 min temporal resolution and a spatial resolution higher than 100 km for more than 45 h even in the daylit atmosphere, which is valuable for comprehensive monitoring throughout all phases during storms and substorms.

UVI is an FUV camera with a 9.9-degree circle field of view (FOV) that acquires images of the aurora oval of N_2_ LBH in the 160–180 nm spectral range at a highly elliptical orbit. A compact telescope is designed for continuous image acquisition using a coaxial four-mirror system with a 65 mm aperture and a focal length of ~103.6 mm. Mirror coatings are designed for selecting the auroral signal and provide short-wavelength cut-off. Cutting off a long wavelength is accomplished via the solar-blinding ICCD and mirrors with lower reflectance. It can guarantee high sensitivity to interesting signals and high rejection of straylight. Zhang et al. [4] summarized the design of UVI. Apart from the optics and opto-mechanical environment of the UVI, the detector is another critical element for imaging performance.

The UVI photon-counting detector was developed through a deep collaboration between CAS and ESA/Photek UK. The UVI detector module is an ICCD (intensified CCD) imager for the FUV regime. This paper analyzes the ICCD module on ground calibration. Instrument-level evaluation can be performed by using the ICCD module characteristics. Following the introduction in Section 1, Section 2 describes the ICCD module design. Section 3 presents the ICCD calibration and test, including the test method, data analysis, and discussion, while assessing the performance of the ICCD assembly. The last section summarizes this work and offers various conclusions.

## 2. Detector Design

**Figure 1:**
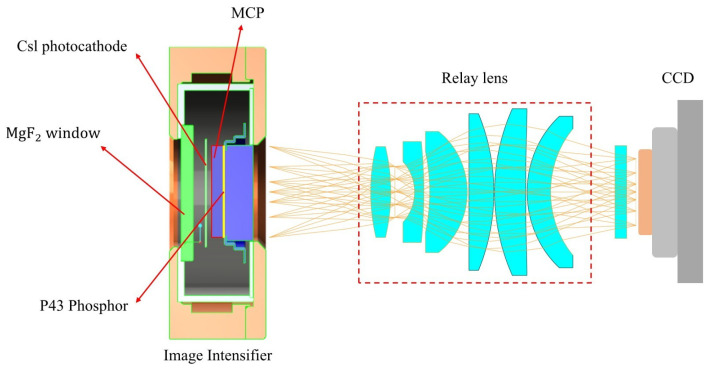
The schematic of imaging configuration of the ICCD assembly.

The UVI detector module is an image intensifier–CCD (ICCD) system, comprising a sealed image intensifier based on single-stage microchannel plate (MCP) detector technology that converts FUV photons into visible-light pulses via relay lens coupling, and then images using a back-illuminated CCD with TEC cooling. The image intensifier is a core assembly that intensifies the incoming UV signal and presents the output photon signal to the image through a relay lens coupled to the CCD detector. Three high voltages are applied to the photocathode, MCP, and phosphor screen, respectively. At the same time, the HV electronic board does not include the ICCD assembly. Figure 1 presents the overall configuration of the ICCD assembly.

Image intensifiers comprise four functional elements: the MgF2 window that provides high transmission at the desired wavelength and high cut-off at short wavelengths (<100 nm) while protecting the intensifier, the solar-blind Cesium Iodide (CsI) photocathode thin layer, which is deposited on the rear end of the MgF_2_ window to provide a longer wavelength cut-off; an incoming photon that creates free electrons; an MCP with 18.1 mm diameter to accelerate the free electron to create the electron cloud; and a P43 phosphor screen with BK7G18 Glass as the output window to convert the multiplied photoelectrons to visible-light emission (peak wavelength is 545 nm). These elements are enclosed by Arathane and Titanium alloy housing with suitable leads for HV encapsulation and electrical connection, respectively. The image intensifier was manufactured at Photek in the UK as part of a collaboration between CAS and ESA/Photek.

In order to operate the image intensifiers, three high-voltage power supplies (HVs), known as HV1, HV2, and HV3, are applied to the image intensifier. HV1 accelerates photoelectrons from the photocathode towards the MCP, HV2 accelerates the secondary electrons through the MCP pore structure, and HV3 accelerates the multiplied electron cloud from the MCP to the phosphor screen, exciting the phosphor photons to visible-light emission. These three HVs can be adjusted to achieve optimal image intensifier performance.

**Figure 2:**
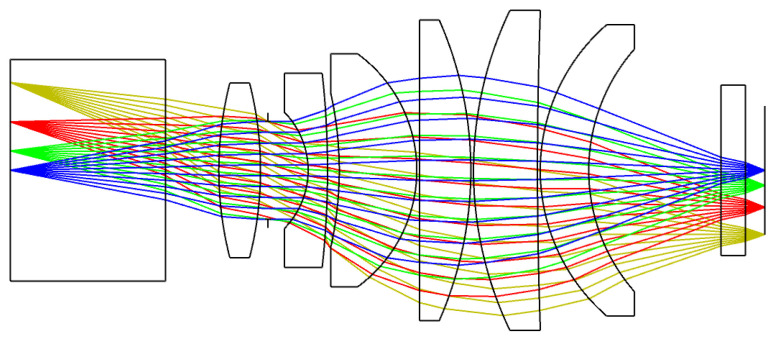
The optical layout of the relay lens system, including the front and rear mirror groups.

Compared with the fiber taper coupling system, high-numerical-aperture coupling lenses are selected based on their alignment feasibility and the requirement for high-quality images. For the relay lens system shown in Figure 2, since the object surface diameter is 18.1 mm and the image plane size is 13.3 mm, a magnification of 0.73 should be considered. Since the size of the numerical aperture determines the efficiency of the collecting light, the numerical aperture should be designed as large as possible. This optical system is a relay system in which the aperture stop is placed between the front and rear mirror groups. The front mirror group concentrates light and changes the propagation direction, while the rear lens group performs the aberration correction. After optimization, the relay lens system provides a magnification of 0.75, a numerical aperture of 0.28, and an MTF greater than 0.7 @ 9.6 lp/mm.

However, the UVI detector employs a readout scheme entirely different from that used in FUSE, GUVI/SSUSI, GOLD, and FY-3/WAI. Double-delay-line and WSZ anode systems read the detectors used on these missions [5,6,7]. At the same time, the UVI detector incorporates a CCD-based readout system using an optical readout imaging scheme, similar to IMAGE/WIC and POLAR/UVI [3,8], and the ICON Far Ultra Violet Imaging Spectrograph [9]. The photoelectrons (generated in and accelerated by the MCP) are converted to light through a phosphor. The photons generated by the phosphor are coupled to the CCD sensor via the relay lens. The E2V CCD 47–20 with 1024 × 1024 pixels is selected. However, the CCD pixels on the chip are binned by 2 × 2 to minimize read noise and the effects of dark current noise. Thus, a 512 × 512 raw image is obtained for data downlink, and the CCD operates at approximately −20 °C.

## 3. ICCD Calibration and Testing

For the FUV instrument including a wide-field imaging camera or a spectrometer, due to its working wavelength being in the vacuum ultraviolet range, ground-based calibration and testing need to be carried out in a vacuum environment [10,11,12]. This means that this is a time-consuming and complex workflow. Before the UVI instrument alignment, a comprehensive test and calibration were performed to evaluate the performance of the ICCD module. The quantum efficiency (Qe) of the imager intensifier, the gain–voltage relationship, dark current and noise, the excess noise factor, the non-linearity, the dynamic range, image distortion, the modulation transfer function (MTF), image uniformity, and the signal-to-noise ratio (SNR) model were evaluated through this test.

### 3.1. QE of the Intensifier

**Figure 3:**
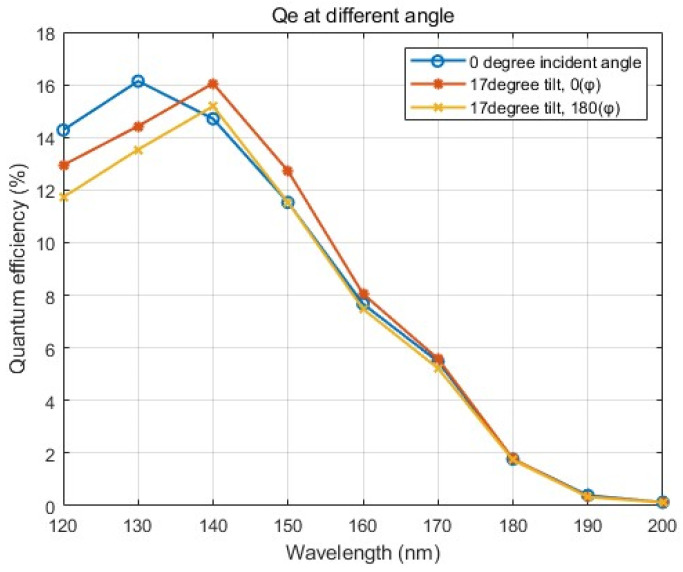
The QE results of the image intensifier at three incident angles. The circles refer to a 0-degree incident angle, while the dots and stars refer to the incident angle of 17 degrees with azimuthal angles of 0 and 180 degrees, respectively.

As a critical parameter for the FUV signal, the QE of the intensifier was immediately measured at Photek UK after sealing the MCP-118 detector. For the image intensifier, a negative HV was applied at the rear end of the MgF2 window to create a field bias between the window and the MCP. The photocathode generates electrons from incident photons traveling through the input window, but not every incident photon can produce a photoelectron, as some photons may be reflected, transmitted, or scattered away. The quantum efficiency of a photocathode also depends on the applied electric field. When an appropriate electric field is applied in front of the MCP input, secondary electrons generated from primary electrons striking the solid inter-channel regions are redirected back toward the channel openings, thereby collecting photoelectrons emitted away from the MCP surface and enhancing the quantum efficiency [13]. The image intensifier sample testing demonstrated a QE enhancement of about 20–30%, similar to the result obtained with the FUV detector of the ICON FUV instrument [14]. The measurements should be performed at multiple incident and azimuthal angles relative to the optical axis to assess the impact of different incident angles. Multiple measurements were performed to assess response stability and improve measurement accuracy. A reference synchrotron source was used as the calibrated source in the absence of drift. A deuterium lamp and an FUV monochromator were used during the measurement to obtain the input signal at 120–200 nm with 10 nm increments. All measurements included a 7 mm thick MgF_2_ input window at a light incident angle of 17 degrees from perpendicular, with azimuthal angles of 0 and 180 degrees, respectively. Figure 3 shows the QE results. The average Qes for three angles range from ∼0.12% to ∼8.16% over a wavelength range of 120 nm to 200 nm. For system performance, the average QE in the desired wavelength region (160–180 nm) is about 3.86%, after removing the influence of the transmittance of the MgF2 window.

### 3.2. Radiant Gain

To reflect the ability of an image intensifier to convert input photons into output photons, radiant gain is defined as the ratio between input photons on the MgF_2_ window and output photons through the BK7G18 Glass window. Photon gain is a hybrid characteristic of the input QE, MCP electron gain, phosphor screen gain, and transmittance of windows. This measurement is for the image intensifier unit performed by ESA/Photek.

**Figure 4:**
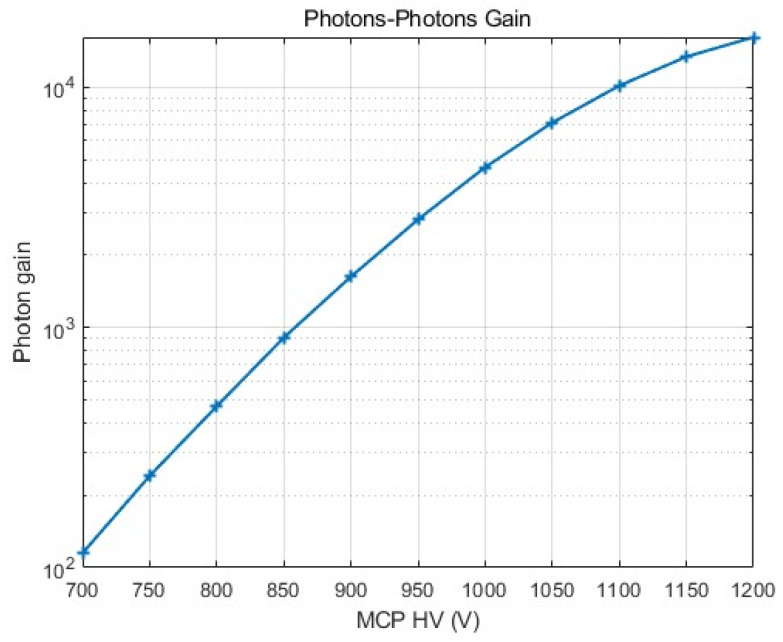
Radiant gain–voltage curve for the image intensifier.

The measurement principle is to use the calibrated FUV photometer to produce a light source that illuminates the image intensifier’s input windows. The known spectral response at a specified wavelength and a photocathode current is measured to calculate the total number of input photons. A calibrated flat-response radiometric sensor and an optometer observe the output phosphor screen at a fixed distance and at a specified wavelength. Given Lambertian output from the phosphor screen, the total power output can be calculated, thereby determining the number of output photons. The measurement is performed at multiple MCP operating voltages, ranging from 700 to 1200 V in 50 V increments, with a typical phosphor screen operating voltage of 5500 V and a photocathode voltage of −290 V. Figure 4 shows the photon gain result. As presented in Figure 4, the gain can be varied from ~10^2^ to ~2 × 10^4^ by adjusting the high voltage on the MCP in the range 700–1200 V. Based on the available signal, a margin can be applied to the MCP’s operating voltage to detect the signal by raising the MCP’s high voltage.

### 3.3. Equivalent Background Illumination

As a core parameter in ICCD performance evaluation, equivalent background illumination (EBI) characterizes the ICCD’s background noise level. It directly impacts the detection sensitivity, SNR, and dynamic range. Reducing the EBI value is key to improving detector performance, especially in extremely low-light applications. The impact of EBI on imaging quality can be effectively controlled by optimizing the CCD’s operating temperature, gain, and integration time.

EBI measurements were performed under high-vacuum conditions and maintained at a specified temperature of 21 ± 1 °C. An FUV monochromator with an FUV light source was used to output incoming light at 160 nm, with an MgF_2_ diffuser and a collimator for further homogenization. A standard detector calibrated by NIST was employed to measure the incident light intensity (I). Subsequently, the photon response (*DN_s_*) and dark background (*DN_d_*) were detected by ICCD at −290 V @ HV1, 950 V @ HV2, and 5500 V @ HV3, while the CCD’s integration time was 2 s. Finally, the following formula was used to calculate the EBI.


(1) $$\mathrm{EBI}=(\frac{{\mathrm{DN}}_{d}}{{\mathrm{DN}}_{d}+{\mathrm{DN}}_{s}})I$$

According to the measurement, the EBI across the entire image area can be approximately calculated using an active area of 7.8 mm × 7.8 mm, yielding 3.697 × 10^5^ photons/s.

### 3.4. Excess Noise Factor

The excess noise factor (ENF) is a dimensionless parameter that quantifies the extra noise generated by the stochastic gain process in an image intensifier. It is a critical metric for evaluating the performance of image intensifiers and ICCDs. ENF characterizes the degradation of the SNR during the amplification of the input photoelectron. Based on the theoretical foundation for the derivation extracted from the mathematical baseline for intensified X-ray imaging systems [15], the formula for the measurement and calculation of the ENF is expressed in Formula (2); the last two terms in the formula represent the contribution of the relay lens used in the test.

(2) $$\mathrm{ENF}=\frac{\Phi_{\mathrm{UV}}A_{\mathrm{is}}t_{\exp}\eta_{\mathrm{pc}}}{{\mathrm{SNR}}^{2}}-\frac{1}{G_{\mathrm{UV}}{\left({\mathrm{NA}}_{I,rl}\right)}^{2}\tau_{\mathrm{rl}}\eta_{\mathrm{is}}}+\frac{1}{G_{\mathrm{UV}}\;}$$

where $\Phi_{\mathrm{UV}}$ is the UV photon fluence rate in the image intensifier; $A_{\mathrm{is}}$ is the image sensor collection area; $t_{\exp}$ is the integration time; $\eta_{\mathrm{pc}}$ is the photocathode QE; $G_{\mathrm{UV}}$ is the intensifier quantum gain; ${\mathrm{NA}}_{I,rl}$ is the numerical aperture of the relay lens (image space); $\tau_{\mathrm{rl}}$ is the transmission of the relay lens; and $\eta_{\mathrm{is}}$ is the QE of the image sensor.

**Figure 5:**
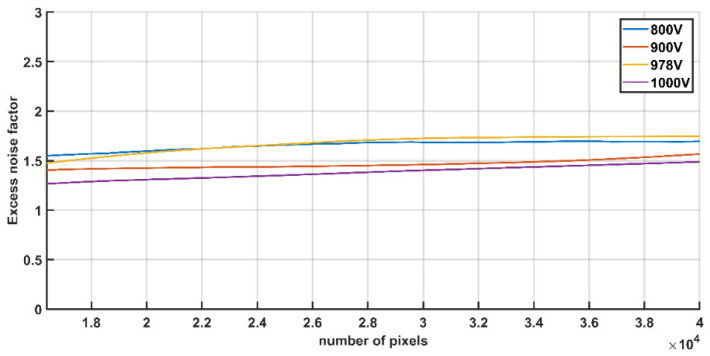
The measurement of the excess noise factor at different HVs for the MCP.

The experiment measures the signal over a fixed area using a fixed photon flux input and a known photon gain for four MCP operating voltages, with a fixed-focal-length camera/lens. To estimate the excess noise factor, the SNR of the image intensifiers is measured under well-controlled illumination conditions at Photek. A dedicated relay lens paired with an image sensor is used. The distances from the phosphor screen to the relay lens, as well as the lens to the image sensor, are fixed to ensure proper focus, with a magnification of 1. The uniform illumination at 170 nm generated from an FUV monochromator with the deuterium lamp is directed onto the image intensifier input window. For each status, 100 frames under illuminated and dark conditions re acquired, respectively. In the data processing, the image sub-region analysis method is adopted. The unreasonable signal regions caused by distorted areas, abnormal noise pixels, and non-uniform illumination during the testing process are identified. After removing these invalid data regions, the remaining active areas are used to calculate the SNR; then, according to Formula (2), the ENF for different operating voltages can be obtained. The image intensifier is operated at a known gain and illuminated by a known photon flux $\Phi_{\mathrm{UV}}$ at a wavelength of 170 nm. Figure 5 shows the measured noise factor for four different operating voltages and different active area sizes ranging from 128 × 128 to 200 × 200 pixels centered in the images, which is at the center of the illuminated parts of the field of view. The excess noise factor varies slightly with different HVs for the MCP. The excess noise factor is between 1.26 and 1.75 within the normal high-voltage range of the MCP. This provides a reference for noise assessment in the instrument-level testing.

### 3.5. Image Distortion

The image distortion of the ICCD is attributed to the image intensifier, relay lens, and CCD detector. Distortion assessment will be conducted after the ICCD alignment. The 160 nm monochromatic light is uniformly illuminated onto the ICCD input window through the diffuser and collimator. The ICCD captures an image of a dedicated pinhole array.

**Figure 6:**
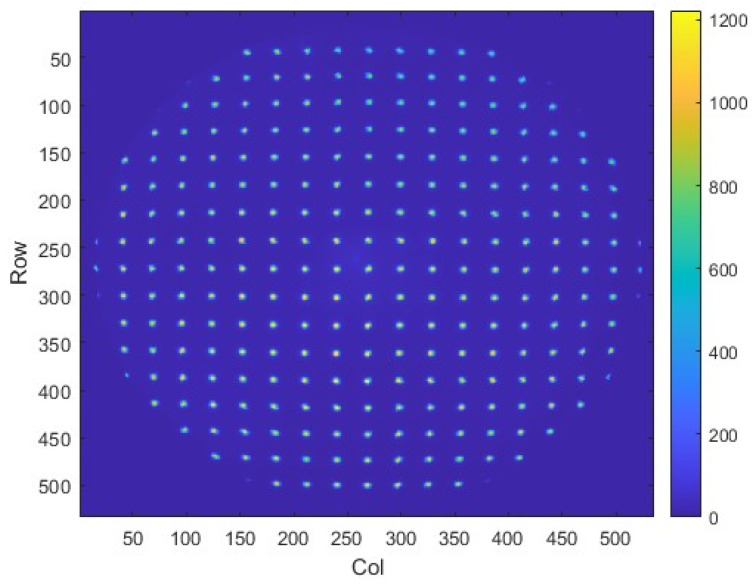
The distorted image of the ICCD using a pinhole array.

**Figure 7:**
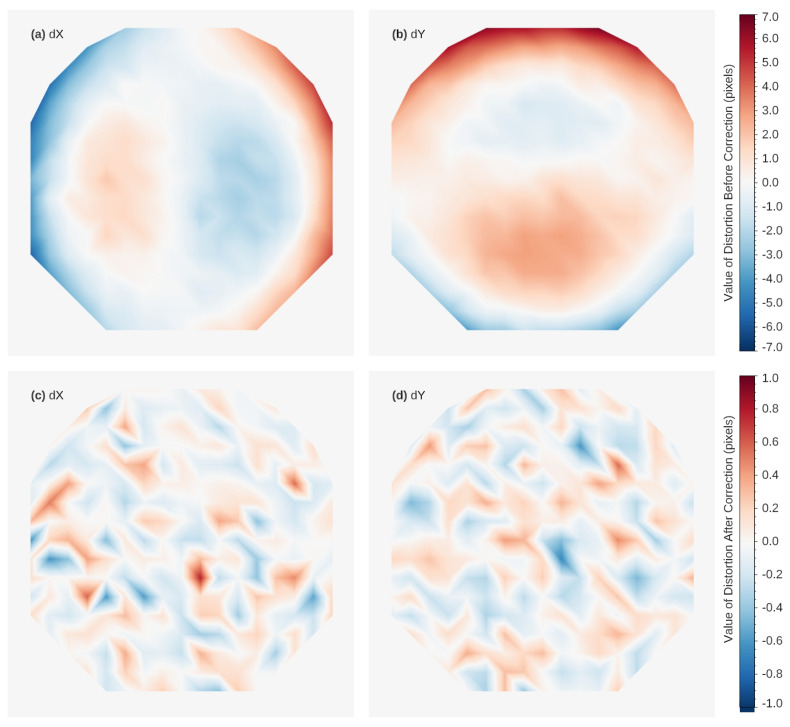
Distributions of the geometric distortion values across the whole image plane. (**a**) Distortion values in X direction (Col in Figure 6) before correction. (**b**) Distortion values in Y direction (Row in Figure 6) before correction. (**c**) Residual of distortion in X direction after correction. (**d**) Residual of distortion in Y direction after correction.

Figure 6 shows the distorted raw image of the ICCD. Compared with the standard grid of the pinhole array, the central area of the image shows no distortion, while the edge areas exhibit slight radial distortion. Based on the raw image, a two-dimensional fourth-order polynomial can be fitted to modify residual distortion, thereby effectively correcting geometric distortion in the image. The distributions of the distortion values before and after correction are shown in Figure 7. It is shown that the residual distortions are less than one pixel.

### 3.6. MTF

Different from conventional CCD and CMOS, the modulation transfer function (MTF) of ICCD is mainly determined by the image intensifier, relay coupling lens, and CCD. While ensuring that the MTF of the image intensifier is as high as possible, the MTF of the ICCD assembly is mainly determined by the performance of the relay coupling lens. Due to the limitation of satellite mounting resources, the volume of UVI is subject to strict constraints. To maximize energy concentration as much as possible, the relay coupling lens requires a large numerical aperture and an extremely small working distance. After optimization, the MTF of the relay lens is greater than 0.7 @ 9.6 lp/mm.

For the UVI instrument, the required spatial resolution is 150 km @ 19Re, which means the IfoV is 0.058° with 6 × 6 binning for the CCD image, and the spatial frequency is 6.4 lp/mm. The design goal, however, is to achieve an IfoV of 0.039° with a 4 × 4 binning of the CCD image, and a spatial frequency of 9.6 lp/mm. To evaluate the spatial resolution level, both the MTF at 9.6 lp/mm and that at 6.4 lp/mm are measured. These MTF measurements provide critical insights into the instrument’s ability to resolve fine structure under different binning configurations, ensuring that it meets the specified spatial resolution requirements for its scientific objects.

MTF tests are performed using the Hg lamp (peak wavelength is 253.7 nm) and a dedicated line pair target in an atmospheric environment. The bar pattern target line width is designed to comply with the UVI instrument’s requirement, which specifies spatial frequencies of 9.6 lp/mm and 6.4 lp/mm for matching to the UVI spatial resolution. The Hg lamp source illuminates the target from behind through a MgF2 diffuser and a standard object lens. Then, the ICCD captures the target image at MCP HV with 950 V. Formula (3) calculates the contrast transfer function (CTF) from the average bright (${\mathrm{DN}}_{\max}$) and average dark (${\mathrm{DN}}_{\min}$) line pairs of the image. At the same time, Formula (4) calculates the MTF. MTF can be measured at five positions across the image, including the center, left, right, top, and bottom at whole image areas, as shown in Table 1. The average MTF values are 0.315 @ 9.6 lp/mm and 0.435 @ 6.4 lp/mm with whole image areas, respectively. This demonstrates that it meets the performance requirement of the SMILE mission.


(3) $$CTF=\frac{\left({\mathrm{DN}}_{\max}-{\mathrm{DN}}_{\min}\right)}{\left({\mathrm{DN}}_{\max}+{\mathrm{DN}}_{\min}\right)}$$

(4) $$\mathrm{MTF}=\mathrm{CTF}\times \frac{\pi }{4}$$

### 3.7. Image Uniformity

According to the ICCD principle, three factors arising from image plane response non-uniformity affect the accuracy of quantitative measurement. Among them, the main factor is the inhomogeneity of the photoelectric response from the image intensifier, which is further reduced by the non-uniformity of the photocathode’s quantum efficiency, the fixed-pattern noise of the MCP, and the brightness inhomogeneity of the phosphor screen and the output window. The other two factors are the relay lens distortion and the CCD region pixel response non-uniformity (PRNU).

**Figure 8:**
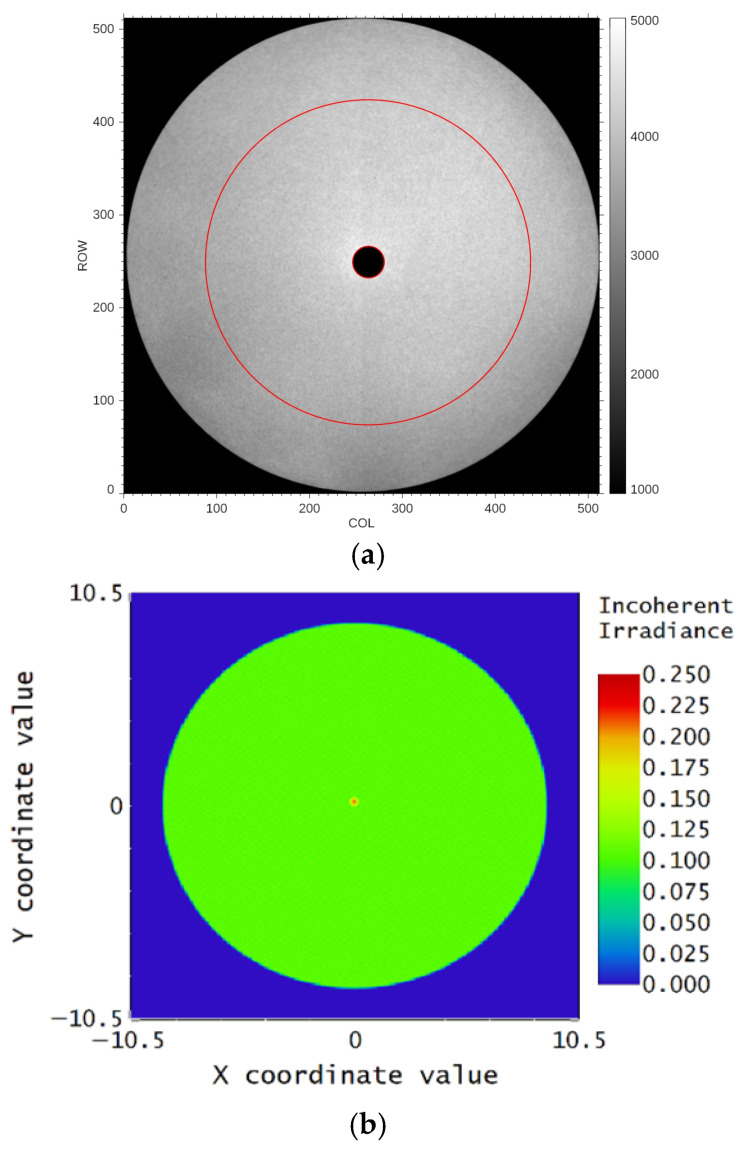
The measurement and simulation for the flat field of the ICCD. (**a**) The measurement result of the flat field for the ICCD. (**b**) The simulation results for the bright spot phenomenon without a mask.

The uniformity of the light source is critical for testing the non-uniformity of the FUV ICCD. To minimize the non-uniformity of the light source, a set of FUV monochromators with a deuterium lamp was employed to generate 160 nm monochromatic light. Then, the collimated and homogenized beam from the FUV collimator and MgF2 diffuser was used to illuminate the ICCD input window. Figure 7a shows the test image of the flat field for the ICCD assembly. The collimated light with a 200 mm diameter is incident on the ICCD focus plane without limiting the incident angle, generating several additional light paths into the center of the ICCD, thereby creating a bright spot (shown by the small circle in Figure 8a). The bright spot will not be observed after integration with the UVI telescope system. Figure 8b shows the simulation results for the bright spot phenomenon, which are consistent with the test result in Figure 7a. The bright spot area of the image is removed during the image uniformity evaluation.

The 60 flat-field frames are summed to suppress random and photon noises. After subtracting the dark background captured with the same HVs and integration time, the non-uniformity can be calculated by the standard deviation-to-mean ratio for specified areas after removing the bright spot. First, the PRNU for the whole active area is evaluated. To reduce the contribution of non-uniform spatial illumination, the PRNU values are computed in four selected windows, including the upper, lower, left, and right semi-circles. As shown in Table 2, the mean PRNU for the whole image area is 8.39%. Then, the PRNU considering loss from vignetting at the edge of the field is also evaluated. The selected area is in the circular ring (the circular ring region is defined by the two red circles in Figure 8a), which corresponds to a field of view about ±3.5° (circle), and the PRNU is 6.15%.

### 3.8. The SNR Model and Non-Linearity

ICCD is a highly sensitive detector that can amplify and image weak signals. Compared with conventional CCD and CMOS detectors, it has more complex noise configurations due to its ability to amplify weak signals [16]. According to the working principle of ICCD, its noise mainly comprises four parts: photon shot noise, image intensifier noise, relay coupling noise, and CCD noise. Among them, photon shot noise is an inherent quantum characteristic of light following a Poisson distribution. Image intensifier noise primarily includes fluctuations in photocathode quantum efficiency, MCP noise, and phosphor screen noise. The random process during photons striking the photocathode and generating photoelectrons, which also follows a Poisson distribution, generates fluctuations in photocathode quantum efficiency. MCP noise, which is the most significant contributor to image intensifier noise, is generated when a photoelectron enters an MCP channel and triggers an avalanche-like secondary electron multiplication through multiple highly random collisions. An excess noise factor describes the additional noise introduced by the MCP. Phosphor screen noise mainly results from fluctuations caused when the electrons bombard the phosphor screen to generate photons. The noises of the CCD camera primarily include dark current and readout noises. In summary, Equation (5) calculates the SNR of ICCD.

(5) $${SNR}_{ICCD}=\frac{{Signal}_{out}}{\sigma_{Total}}=\frac{S_{P}\;\cdot QE_{PC}\;\cdot G_{int\;}\cdot CE\cdot \;QE_{CCD}}{\sqrt{S_{P}\;\cdot QE_{PC}\;\cdot {\;G}_{int\;}\cdot ENF\cdot CE\cdot \;QE_{CCD}+\sigma_{dark}^{2}+{\;\sigma }_{read}^{2}}}$$

where $S_{P}$ is the incoming signal, $QE_{PC}$ is the photocathode quantum efficiency, $G_{int}$ is the gain including the MCP and phosphor screen, CE is the relay lens coupling efficiency, $QE_{CCD}$ is the CCD’s quantum efficiency, ENF is the excess noise factor of the image intensifier, and $\sigma_{dark}^{2}$ and $\sigma_{read}^{2}$ indicate the dark current and read noises, respectively.

**Figure 9:**
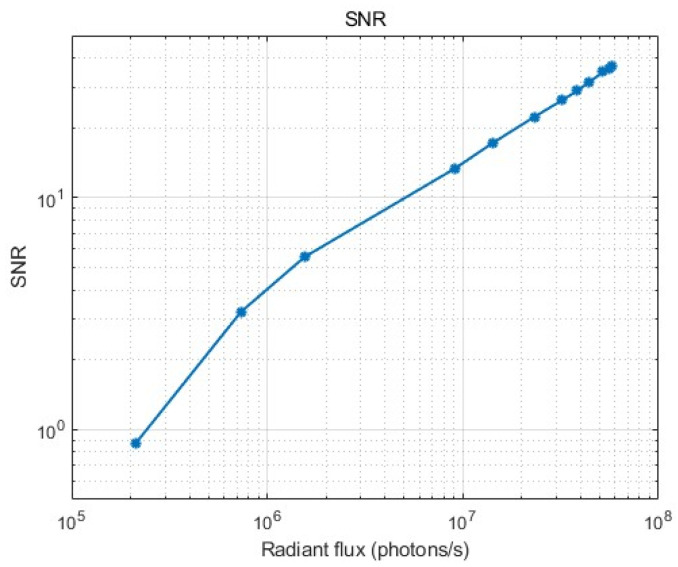
The SNR of the ICCD versus the radiant flux. HVs of −290 V, 1000 V, and 5500 V were employed for the photocathode, MCP, and phosphor screen, respectively. The integration time was 3 s, and the image cadence was 1 min.

An FUV monochromator with a collimator was used to measure the SNR of the ICCD detector at 160 nm, obtaining a collimated beam, while a silicon photodiode AXUV-100G developed by IRD US was used to measure the input light. The photodiode was calibrated by NIST. The image intensifier, with applied voltages of −290 V, 1000 V, and 5500 V, was employed for the photocathode, MCP, and phosphor screen, respectively. The operating temperature for the CCD was −20 °C ± 0.5 °C, which is the same as in orbit; the integrating time was 3 s; and the image cadence was 1 min. Figure 8 shows the SNR versus the radiant flux for ICCD. Due to the limitations of the deuterium lamp, higher signals could not be measured. As shown in Figure 9, the SNR has a non-linear relationship with the input light intensity. It can be observed that the ICCD demonstrates detection capability in regions with a relatively weak signal. However, as the incident light intensity increases, the excess noise factor of the image intensifier becomes the primary factor affecting SNR.

In a similar setup, the non-linearity of the ICCD was assessed by varying the intensity of the incident light while maintaining the ICCD parameters at the same values as those used for the SNR testing. The photodiode AXUV-100G calibrated by NIST was used to record the intensity of incident light. Equation (6) defines the non-linearity:

(6) $$NonLinear=\frac{\left(DN_{\mathrm{test}}-DN_{\mathrm{fit}}\right)}{DN_{fit}}\times 100\%$$

**Figure 10:**
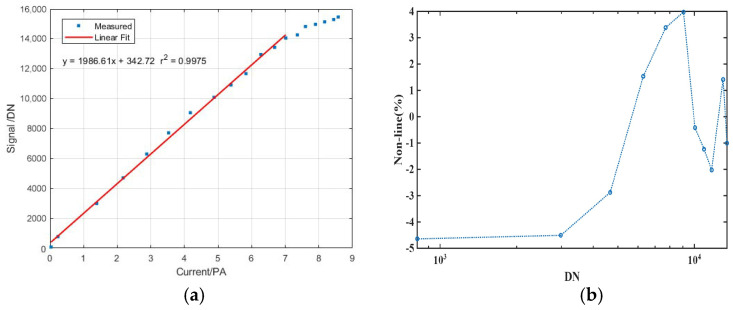

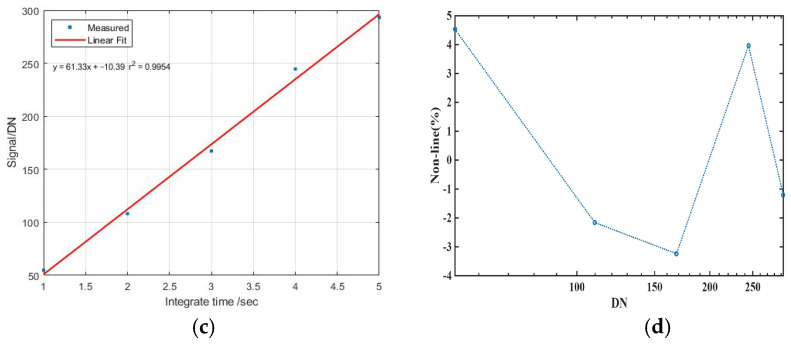
Comparison between the measured signals and linearly fitted signals. (**a**) The measured and linearly fitted signals versus the intensity of the incident light. (**b**) The non-linearity of samples vs. signal level in (**a**). (**c**) The measured and linearly fitted signals versus the integration time at a specified light intensity and (**d**) The non-linearity of samples vs. signal level for (**c**).

Figure 10 describes the non-linearity of all samples. As shown in Figure 10a, the deviation between the measured and linearly fitted signals, plotted against the intensity of the incident light, indicates a non-linear relationship between the signal and current, and the non-linearity of other samples vs. the signal level, except for the sample at minimum signal, which is shown in Figure 10b. In addition, due to the limitation of the light source, the non-linearity at the lower signal level can be measured by varying the integration time at a specified light intensity, as shown in Figure 10c, and the non-linearity of all samples vs. signal level is shown in Figure 10d. The non-linearity analysis indicates that the non-linear relation can be approximated with a linear relationship. In the dynamic range of 51–14270 DN, the deviation between the measured and linearly fitted values is below 5%, indicating a sufficiently high detection range of the ICCD for aurora.

## 4. Conclusions

The UVI is an instrument designed for continuous auroral monitoring, capable of operating for more than 45 h with a maximum temporal resolution of 1 min. It employs a highly sensitive, solar-blind, and intensified CCD. The ICCD comprises a sealed image intensifier based on a single-stage MCP intensifier with an 18.1 mm active area, relay coupling lens, and CCD. Detailed tests were performed on the ICCD to comprehensively evaluate its characteristics and performance, including the quantum efficiency, gain, noise, and imaging quality. Accordingly, an SNR model was established. These results provide an important basis for UVI-level performance. An instrument-level calibration test was completed after integration with the telescope system. It was demonstrated that the UVI’s radiation sensitivity is better than 100 R at a spatial resolution of 0.04° and a maximum temporal resolution of 1 min [4].

Due to its excellent noise suppression capabilities and good imaging quality, this high-performance ICCD can be employed for future detection of deep space auroras, such as those of Jupiter and Saturn, as well as other weak vacuum ultraviolet signals [17,18].

## Figures and Tables

**Table 1:** MTFs at various positions at a spatial frequency of 9.6 lp/mm and 6.4 lp/mm.

Field Location# (Row, Column)	MTF @ 9.6 lp/mm	MTF @ 6.4 lp/mm
No. 1 (256, 253)	0.336	0.456
No. 2 (256, 20)	0.318	0.432
No. 3 (256, 493)	0.298	0.398
No. 4 (25, 253)	0.315	0.454
No. 5 (490, 253)	0.308	0.437
Average	0.315	0.435

**Table 2:** The PRNU test for the whole image area.

Local Area (Semi-Circle)	PRNU
Upper	9.15%
Lower	7.31%
Left	8.43%
Right	8.68%
Average	8.39%

## Data Availability

The data presented in this study are available on request from the author.

## Abbreviations

The following abbreviations are used in this manuscript:

DN	Digital Number
FUV	Far Ultraviolet
ICCD	Intensifier Charge-Coupled Device
LBH	Lyman–Birge–Hopfield
MCP	Micro Channel Plate
UVI	Ultraviolet Image
SMILE	Solar Wind Magnetosphere Ionosphere Link Explorer
